# A Variational Formulation for Irreversible Thermodynamics with Path Dependence

**DOI:** 10.3390/e28010094

**Published:** 2026-01-13

**Authors:** Huilong Ren

**Affiliations:** State Key Laboratory of Disaster Reduction in Civil Engineering, College of Civil Engineering, Tongji University, Shanghai 200092, China; hlren@tongji.edu.cn

**Keywords:** variational thermodynamics, entropy production, irreversible processes, path-dependent action, continuum thermodynamics

## Abstract

This work introduces a path-dependent energy Lagrangian for irreversible thermomechanics that embeds heat and entropy accounting directly into the action. The formulation requires neither Lagrange multipliers nor Rayleigh potentials. An explicit θs term enforces Helmholtz conjugacy and positive heat capacity; writing heat as a divergence produces the natural flux; nonnegative dissipative productions are collected in a single modular term; and a history integral supplies an upper-limit variation that converts instantaneous power into entropy production. Stationarity yields the standard field equations together with a global entropy balance and a channel-wise power identity by placing each production once in entropy and once, with opposite sign, in its own channel. Classical closures—including Fourier and non-Fourier heat conduction, diffusion, and viscous mechanics—arise as special cases of the same functional. Compact examples show how the framework provides a unified action, a single entropy audit, and consistent positive production across coupled dissipative mechanisms.

## 1. Introduction

Continuum thermodynamics rests on two macroscopic statements: energy is conserved and entropy is produced. From Carnot’s analysis of the motive power of fire and early formulations of the Second Law by Clausius and Thomson [1,2,3] to Gibbs’ equilibrium thermodynamics [4] and rational continuum theories [5,6,7], these principles have been encoded in local balance laws and constitutive restrictions. Classical expositions [8,9] treat entropy production by postulating an energy balance, an entropy inequality and material response functions that guarantee nonnegative production. In mechanics, the Coleman–Noll procedure and its successors [10,11,12] provide a systematic route from free energies and internal variables to thermodynamically admissible field equations. While effective, this framework keeps irreversibility outside any unified variational structure: energy and entropy are linked through inequalities rather than via a single action.

At the same time, variational principles form the backbone of reversible mechanics. From Lagrange and Hamilton to Landau and Arnol’d [13,14,15,16], conservative systems are derived from an action whose stationarity yields the Euler–Lagrange equations and, via Noether’s theorem, conservation laws [17]. In continuum mechanics these ideas underpin modern formulations and structure-preserving discretizations [18,19]. However, when heat conduction, diffusion, viscosity, or chemical reactions are present, the usual practice is to append phenomenological terms to the balance laws. These include Fourier and Fick laws, viscous stresses, and reaction kinetics [9,20,21]. In this setting, the action is typically kept for the reversible part only. Alternatively, it is extended in an ad hoc way using Lagrange multipliers or Rayleigh potentials.

Several influential frameworks have attempted to bring irreversibility closer to a variational or geometric setting. Onsager’s theory relates thermodynamic forces and fluxes via symmetric positive semidefinite matrices and explains near-equilibrium transport through a quadratic dissipation functional [9,22,23,24]. GENERIC and metriplectic dynamics combine an antisymmetric (Hamiltonian) bracket with a symmetric dissipative bracket. Degeneracy conditions then enforce energy conservation and nonnegative entropy production [25,26,27,28]. In parallel, the energy dissipation principle and gradient flow theory interpret irreversible evolution as the steepest descent of an energy in problem-dependent metrics; this viewpoint has been rigorously developed in Wasserstein and related settings [29,30,31,32,33] and applied to reaction–diffusion and drift–diffusion systems [32]. More recently, contact-geometric formulations have offered a differential-geometric language for nonequilibrium systems and their integrators [34,35]. Despite their strengths, these approaches typically require prescribed metrics or brackets and enforce the entropy inequality through external conditions. As a result, it is not straightforward to incorporate multiple dissipative mechanisms within a single, compact action.

Irreversibility is also naturally tied to history. Stochastic thermodynamics employs path-dependent functionals to quantify work, heat and entropy at the trajectory level, leading to fluctuation theorems such as Jarzynski’s equality and related results [36,37]. In macroscale continuum models, however, path dependence is usually encoded through internal variables and memory kernels [38] rather than by embedding entropy accounting directly into an action. Extended heat conduction theories with finite propagation speed—such as Cattaneo–Vernotte, Guyer–Krumhansl, and modern nanoscale variants [39,40,41,42,43,44]—further complicate the variational picture. Fluxes become dynamic and introduce relaxation times, while entropy production must remain nonnegative across multiple coupled channels.

For coupled thermo-mechanical and transport problems, this separation between reversible variational principles and irreversible balance-plus-inequality formalisms has practical consequences. As models grow to include diffusion–reaction networks, viscous mechanics, electrochemical Joule heating and non-Fourier conduction, it becomes harder to maintain a consistent entropy audit, avoid double counting of dissipative power and design time discretizations that respect both the First and Second Laws [7,12]. In addition, recent Entropy papers have revisited thermodynamic stability principles and variational viewpoints for irreversible processes [45,46]. What is missing is a concise continuum formulation in which both the standard field equations and an entropy/heat balance with nonnegative production follow from a single variational principle. Such a formulation should clearly separate reversible storage from irreversible dissipation and avoid auxiliary multipliers, Rayleigh potentials, or bespoke bracket constructions.

The present work introduces such a formulation in the form of a path-dependent energy Lagrangian (PDEL) for irreversible thermodynamics. The key idea is to augment a conventional Lagrangian with an explicit thermal storage term and a history integral. The history term accumulates heat-flux divergence, external heating, and nonnegative productions across dissipative channels. Stationarity with respect to temperature enforces Helmholtz conjugacy and a positive heat capacity. A tangential (time-relabeling) variation in the history term turns instantaneous power into entropy production and yields a Clausius-type entropy balance without Lagrange multipliers. A weak power balance closes each rate–co-force channel—and gives a single-entry identity. Each channel’s production enters the entropy balance with a positive sign and enters its own evolution equation with the opposite sign, preventing overcounting and fixing the sign structure at the action level.

Within this framework, classical closures appear as specializations rather than independent postulates. Fourier and Cattaneo–Vernotte heat conduction, including finite-speed and relaxation effects, are recovered by appropriate choices of flux variables and co-forces [20,38,39,40,41]. Diffusion and reaction kinetics in the sense of nonequilibrium thermodynamics [9] emerge from Onsager-symmetric mobilities and affinities with nonnegative quadratic production [9,22,32]. Viscous mechanics and electrochemical Joule heating enter as additional channels whose productions share the same entropy audit. At a structural level, identifying rates with fluxes and co-forces with thermodynamic forces recovers Onsager’s linear relations [22,23,24]. Convex dissipation potentials reproduce the energy–dissipation principle and metric gradient flows [29,30,31,32,33]. Symmetric mobility operators then align with the dissipative sector of GENERIC/metriplectic dynamics [25,26,27,28] and remain compatible with contact-geometric extensions [34,35].

Thus, this paper proposes a compact, path-dependent variational formulation that internalizes entropy and heat accounting into the action and supplies a modular channel architecture for irreversible processes. The goal is not to replace existing geometric or stochastic frameworks, but to provide a continuum-level tool that retains the simplicity of classical Lagrangians while enforcing thermodynamic admissibility and single-entry energy accounting by construction. The formulation is demonstrated on heat conduction, diffusion, chemical reactions and electrochemical couplings, and its relation to Onsager theory and gradient-flow/EDP structures and GENERIC is made explicit. The development is intended for smooth continuum fields in standard settings of continuum thermomechanics. Discontinuous solutions (e.g., shocks), measure-valued limits, and strongly nonlocal interactions are outside the present scope and would require additional analytical and modeling ingredients.

The remainder of the paper is organized as follows: Section 2 develops the path-dependent variational formulation, including the action and stationarity rules, establishes the entropy balance and the power identity. Section 3 applies PDEL to a one-degree-of-freedom (1-DOF) viscous oscillator, presents canonical recoveries, and offers thermodynamic demonstrations of multiphysics phenomena. Section 4 situates PDEL within established frameworks by providing explicit mappings to Onsager’s theory, EDP, and GENERIC. Section 5 concludes with remarks and an outlook.

## 2. Path-Dependent Variational Formulation

### 2.1. Motivation and Construction of the Action

Classical variational principles produce the reversible field equations succinctly, but irreversible effects (heat conduction, diffusion, viscosity, and reactions) are often appended by Lagrange multipliers, Rayleigh potentials, or bracket structures [5,6,8,9,12,25,27,28,47,48]. We seek a simple alternative in which heat/entropy accounting and dissipative productions are internal to a single action, with sign placement fixed by the variational calculus rather than by auxiliary inequalities.

The formulation is constructed on a fixed reference domain $\Omega \subset R^{d}$ with Lipschitz boundary ∂Ω and outward unit normal n. Time varies in a closed interval $\left[t_{0},T\right]$. For any time-dependent field X(·,t), a superposed dot denotes the material time derivative $\dot{X}:=\partial_{t}X$. Spatial derivatives are taken with respect to the reference coordinate and denoted by ∇(·). Inner products are written with “·” for vectors and “:” for second-order tensors (Frobenius product).

The primary thermomechanical states consist of

$$\begin{array}{c} Z\left(X,t\right)\in R^{m}\;(\mathrm{configuration}/\mathrm{internal}\;\mathrm{variables}/\mathrm{concentrations}),\\ \theta (X,t)>0\;(\mathrm{absolute}\;\mathrm{temperature}),s(X,t)\;(\mathrm{entropy}\;\mathrm{density}). \end{array}$$

Unless otherwise stated, it is assumed that $Z,\theta \in C^{1}\left(\left[t_{0},T\right];H^{1}\left(\Omega \right)\right)$ and use virtual variations δZ,δθ,δs that are compactly supported in $\left(t_{0},T\right)$ with $\delta Z\left(t_{0}\right)=\delta Z\left(T\right)=0$; the past history will be frozen in the sense made precise below.

The kinetic energy density is

$$K\left(Z,\dot{Z}\right)=\frac{1}{2}\;{\dot{Z}}^{⊤}M\left(Z\right)\dot{Z},$$

with M(Z) symmetric positive definite, and the Helmholtz free-energy density is ϕ(θ,Z).

The action is postulated as

(1) $$S\left[Z,\theta ,s\right]=\int_{t_{0}}^{T}\int_{\Omega }\left\{\;K\left(Z,\dot{Z}\right)-\phi \left(\theta ,Z\right)-\theta \;s-\int_{t_{0}}^{t}D\left(X,\tau \right)\;d\tau \;\right\}\;dX\;dt,$$

with a history integrand *D* that aggregates divergence heat, external sources, and channel productions,

(2) $$D:=\nabla \cdot q_{0}-r_{0}-\sum_{\alpha }\Phi_{\alpha },\;\Phi_{\alpha }\;=Y_{\alpha }\;{\dot{Z}}_{\alpha }\geq 0.$$

Here $q_{0}\left(X,t\right)$ is the reference heat flux and $r_{0}\left(X,t\right)$ a volumetric heat source. In applications one often adopts Fourier’s law $q_{0}=-k\left(\theta ,Z,\nabla \theta ,\ldots \right)\nabla \theta$ with k≥0, or treats $q_{0}$ as a dynamic flux variable in non-Fourier models such as Cattaneo–Vernotte type theories [39,40,44]. The associated entropy flux will later be identified as $J_{s}=q_{0}/\theta$ when the Clausius balance is derived.

Irreversible processes are organized into channels indexed by α, each described by a rate–co-force pair $\left({\dot{Z}}_{\alpha },Y_{\alpha }\right)$. The quantity

$$\Phi_{\alpha }=Y_{\alpha }\;{\dot{Z}}_{\alpha }$$

is the instantaneous production (power density) in channel α. Positivity $\Phi_{\alpha }\geq 0$ is enforced at the constitutive level (e.g., by positive semidefinite mobilities) [9,22,23,24] and will translate directly into nonnegative entropy production in the variational theory. Typical examples include viscous mechanics, multicomponent diffusion, chemical reactions and Joule conduction [6,9].

In contrast to traditional (reversible) Lagrangians, Equation (1) departs structurally by embedding thermodynamic accounting directly into the action. First, the explicit storage block −ϕ(·,θ)−θs makes stationarity in θ enforce the Helmholtz conjugacy $s=-\partial_{\theta }\phi$ without auxiliary multipliers. Second, heat exchange is represented through $D_{\mathrm{heat}}:=\nabla \;\cdot q_{0}$ and irreversible effects are collected in a single dissipation density *D* via nonnegative channel powers $\Phi_{\alpha }\geq 0$, which fixes sign conventions and prevents double counting. Third, the upper-limit history term $\int_{t_{0}}^{t}D\left(\tau \right)\;d\tau$ ensures that a tangential (time-relabeling) variation converts instantaneous power into entropy production, yielding the Clausius-type entropy/heat balance within the same variational statement.

Path-dependent functionals are also central in stochastic thermodynamics and nonequilibrium statistical mechanics, where they typically quantify trajectory-level work/heat fluctuations and underpin fluctuation relations in probabilistic dynamics [36,37,49,50,51]. Here, by contrast, the path dependence in Equation (1) is deterministic and continuum-level: it is introduced to produce, by stationarity, both the field equations and a channel-wise entropy/heat audit. In particular, the channel closure (weak power balance) yields a single-entry identity in which each $\Phi_{\alpha }$ appears once in the entropy/heat balance and once with the opposite sign in its own evolution equation, so the audit is enforced at the level of the action rather than by external degeneracy conditions.

It is emphasized that the present formulation does not claim new material physics in the absence of new constitutive assumptions: if the same free energy, mobilities, and production laws are adopted, the resulting field equations coincide with established continuum models. The path-dependent action imposes explicit constraints on how irreversible power is deposited into the entropy/heat balance and yields a single-entry accounting by stationarity, thereby fixing sign conventions and preventing omission or multiple counting in coupled settings. This construction is directly checkable at the continuous level and provides a clean target for structure-preserving discretizations, where discrete updates can be designed to reproduce nonnegative production across multiple dissipative channels.

### 2.2. Variations with Frozen History and the Upper-Limit Rule

In the variational setting the virtual variations in the present state are allowed while the past history are fixed,

δZ(τ)=0, δs(τ)=0 for all τ<t.

Thus a history functional of the form

$$J\left(t\right)=\int_{t_{0}}^{t}D\left(\tau \right)\;d\tau$$

responds only through its moving upper limit. Assume $D\in L^{1}\left(t_{0},T\right)$ and $s\left(\cdot \right),Z_{\alpha }\left(\cdot \right)$ absolutely continuous in *t*. Then *J* is absolutely continuous with $J_{t}=D$ a.e., and the standard upper-limit rule gives

(3) $$\delta (\int_{t_{0}}^{t}D\;d\tau )=D\;\delta t.$$

There is no contribution from the frozen past; variations differentiate only through the instantaneous upper limit. This is the mechanism by which *instantaneous power* in *D* enters the entropy/heat balance, while the remaining variations act only on the reversible block K−ϕ and on −θs.

### 2.3. Variation in Temperature: Conjugacy and Heat Capacity

Because the history contribution is frozen except for the upper limit, the temperature variation acts only on the reversible terms −ϕ(θ,Z) and −θs. Holding $Z,\dot{Z},s$ fixed,

(4) $$\begin{array}{cc} \delta S{|}_{\delta \theta }\; & =\int_{t_{0}}^{T}\int_{\Omega }\left(-\;\partial_{\theta }\phi \left(\theta ,Z\right)\;\delta \theta -s\;\delta \theta \right)\;dX\;dt\\ \; & =\int_{t_{0}}^{T}\int_{\Omega }\left(-\;\partial_{\theta }\phi \left(\theta ,Z\right)-s\right)\delta \theta \;dX\;dt. \end{array}$$

Stationarity for arbitrary δθ gives the Helmholtz conjugacy

(5) $$s=-\;\partial_{\theta }\phi \left(\theta ,Z\right).$$

Differentiating Equation (5) with respect to θ gives the heat capacity

(6) $$C\left(\theta ,Z\right):=-\theta \;\partial_{\theta \theta }\phi \left(\theta ,Z\right)\geq 0,$$

consistent with classical thermodynamics [6,8].

### 2.4. Upper-Limit Variation: Entropy/Heat Balance

Let $J\left(t\right)=\int_{t_{0}}^{t}D\left(X,\tau \right)\;d\tau$. A time relabeling t↦t+εη(t) with compactly supported η induces a variation δt=η(t), and by Equation (3),

δJ=Dδt.

All other terms in the action contribute through total time derivatives. For the thermal storage term −θs,

(7) $$\delta \left(-\theta s\right)=-\;\frac{d}{dt}\left(\theta s\right)\;\delta t=-\left(\dot{\theta }\;s+\theta \;\dot{s}\right)\;\delta t=-\delta \theta \;s-\theta \dot{s}\delta t.$$

Collecting the δt contributions yields

(8) $$\delta S=\int_{t_{0}}^{T}\int_{\Omega }\left(-D-\theta \;\dot{s}\right)\;\delta t\;dX\;dt.$$

Stationarity for arbitrary δt gives

(9) $$\theta \;\dot{s}+D=0,$$

that is,

(10) $$\theta \;\dot{s}+\nabla \cdot q_{0}-\sum_{\alpha }\Phi_{\alpha }=r_{0}.$$

Equation (10) is the local entropy/heat balance: each production $\Phi_{\alpha }$ enters with a positive sign due to the upper-limit rule, while divergence of heat flux and external sources appear with their usual signs [6,9]. Defining $J_{s}=q_{0}/\theta$ gives the Clausius form

(11) $$\dot{s}+\nabla \cdot \left(\frac{q_{0}}{\theta }\right)=q_{0}\cdot \nabla \left(\frac{1}{\theta }\right)+\frac{1}{\theta }\sum_{\alpha }\Phi_{\alpha }+\frac{r_{0}}{\theta },$$

whose right-hand side is the entropy production density σ≥0 under standard closures.

### 2.5. Variation in *Z*: Reversible Residual and Weak Power Balance

Because the history is frozen except for the upper limit, variations in Z act only on the reversible block K−ϕ. For state Z with mass *M*,

(12) $$\begin{array}{cc} \delta \int_{t_{0}}^{T}\int_{\Omega }K\; & =\int_{t_{0}}^{T}\int_{\Omega }M\;\dot{Z}\;\delta \dot{Z}=-\int_{t_{0}}^{T}\int_{\Omega }M\;\ddot{Z}\;\delta Z, \end{array}$$

(13) $$\begin{array}{cc} \delta \int_{\Omega }\phi \; & =\int_{\Omega }\partial_{Z}\phi \;\delta Z. \end{array}$$

Thus the reversible Euler–Lagrange residual is

(14) $$E:=M\;\ddot{Z}+\partial_{Z}\phi \left(\theta ,Z\right),\;\delta \int \left(K-\phi \right)=-\int E\;\delta Z.$$

Dissipation is introduced by a weak power balance: For any admissible channel virtual rate $v_{\alpha }$,

(15) $$\sum_{\alpha }\int_{\Omega }E_{\alpha }\;v_{\alpha }+\sum_{\alpha }\int_{\Omega }Y_{\alpha }\;v_{\alpha }=0,$$

giving the channel equation $E_{\alpha }+Y_{\alpha }=0$. Testing with $v_{\alpha }={\dot{Z}}_{\alpha }$ yields the *power identity*

(16) $$E_{\alpha }\;{\dot{Z}}_{\alpha }+\Phi_{\alpha }=0,\;\Phi_{\alpha }=Y_{\alpha }\;{\dot{Z}}_{\alpha }\geq 0.$$

The same production that enters Equation (10) positively appears negatively when the channel is tested, ensuring consistent energy accounting and preventing overcounting.

### 2.6. Energy Balance and Reversible Couplings

Define the total storage e:=K+ϕ+θs. Using $s=-\partial_{\theta }\phi$ and the chain rule,

(17) $$\dot{e}=\dot{K}+\partial_{Z}\phi :\dot{Z}+\theta \;\dot{s}.$$

From Equation (16) with a sum over channels, $E:\dot{Z}+\sum_{\alpha }\Phi_{\alpha }=0$, i.e.,

(18) $$\dot{K}+\partial_{Z}\phi :\dot{Z}+\sum_{\alpha }\Phi_{\alpha }=0.$$

Eliminating $\dot{K}+\partial_{Z}\phi :\dot{Z}$ between Equations (17) and (18), and using Equation (10) gives the local First Law

(19) $$\dot{e}=-\;\nabla \cdot q_{0}+r_{0}.$$

**Proposition.** Suppose a model satisfies the local First Law in Equation (19) with e=ϕ+θs, and the Clausius/entropy balance in Equation (10) with $\Phi_{\alpha }\geq 0$. Then the action by Equation (1) together with the upper-limit rule and the weak power balance recovers (i) $s=-\partial_{\theta }\phi$ from δθ, (ii) the entropy/heat balance from tangential variation, and (iii) the channel equation and power identity $E_{\alpha }\;{\dot{Z}}_{\alpha }+\Phi_{\alpha }=0$.

**Remark.** If $\partial_{\theta Z}\phi \neq 0$, then the cross term $-\theta \;\partial_{\theta Z}\phi :\dot{Z}$ appears in $\theta \dot{s}$ and reorganizes with $\partial_{Z}\phi :\dot{Z}$ into the reversible rate of ϕ+θs; it does not contribute to the entropy production side of Equation (11) [6]. For Fourier $q_{0}=-k\nabla \theta$ with k≥0, Equation (10) reduces to a standard temperature equation with sources $\sum_{\alpha }\Phi_{\alpha }$.

## 3. Applications and Generalization

This section illustrates how the proposed PDEL serves as a practical template for building coupled irreversible continuum models. The guiding rule is modular: each dissipative mechanism is introduced as a rate–co-force channel with a nonnegative production, and channels are combined additively within a single action, yielding a consistent entropy/heat audit by stationarity.

### 3.1. 1-DOF Viscous Oscillator (No Conduction)

Let u(t) be the displacement, θ>0, and *s* the entropy density. Choose the viscous pairing $Y=c\;\dot{u}$, so the channel power is $\Phi =Y\;\dot{u}=c\;{\dot{u}}^{2}\geq 0$, hence D=−Φ. To avoid the trivial s=0 case, take the free energy in the split form

$$\phi \left(\theta ,u\right)=\frac{1}{2}ku^{2}+a\left(\theta \right),$$

where a(θ) is concave in θ (so that $C\left(\theta \right)=-\theta a^{\prime \prime }\left(\theta \right)\geq 0$). For a constant heat capacity $c_{v}>0$, a convenient choice is

$$a\left(\theta \right)=c_{v}\left(\theta -\theta_{\mathrm{ref}}-\theta \ln\left(\theta /\theta_{\mathrm{ref}}\right)\right),$$

which gives $s\left(\theta \right)=-a^{\prime }\left(\theta \right)=c_{v}\ln\left(\theta /\theta_{\mathrm{ref}}\right)$ and $C\left(\theta \right)=-\theta a^{\prime \prime }\left(\theta \right)=c_{v}$.

The PDEL action reduces to

$$S=\int_{t_{0}}^{T}\left\{\frac{1}{2}m{\dot{u}}^{2}-\phi \left(\theta ,u\right)-\theta s-\int_{t_{0}}^{t}D\left(\tau \right)\;d\tau \right\}\;dt.$$

Stationarity yields, in order (i) δθ: $s=-\partial_{\theta }\phi =-a^{\prime }\left(\theta \right)$; (ii) tangential variation: $\theta \;\dot{s}+D=0\Rightarrow \theta \dot{s}=c\;{\dot{u}}^{2}\geq 0$; (iii) δu: using the power identity, the path term contributes −Yδu, while the reversible part contributes $-(m\ddot{u}+\partial_{u}\phi )\;\delta u$; hence E+Y=0 with $E=m\ddot{u}+\partial_{u}\phi$ and $Y=c\dot{u}$. Therefore

$$\;m\ddot{u}+c\dot{u}+\partial_{u}\phi \left(\theta ,u\right)=0\;,$$

which is exactly the governing equation with damping but derived from PDEL using variational principle.

With the split $\phi \left(\theta ,u\right)=\frac{1}{2}ku^{2}+a\left(\theta \right)$, the mechanical equation becomes

$$m\;\ddot{u}+c\;\dot{u}+k\;u=0,$$

and the thermal/entropy audit reads

$$\;\theta \;\dot{s}=c\;{\dot{u}}^{2}\;,\;e.g.,\;\mathrm{for}\;s\left(\theta \right)=c_{v}\ln\left(\theta /\theta_{\mathrm{ref}}\right)\Rightarrow c_{v}\;\dot{\theta }=c\;{\dot{u}}^{2}.$$

This verifies single-accounting: $d(\frac{1}{2}m{\dot{u}}^{2}+\phi )/dt=-\Phi$ while $\theta \dot{s}=+\Phi$. A practical interpretation of the single-entry accounting and its implications for coupled modeling and discretization is summarized in Section 3.5.

### 3.2. Canonical Recoveries and Equivalences

The PDEL is agnostic to the choice of thermodynamic potential. This section shows, in a self-contained way, how the standard identities of equilibrium thermodynamics are recovered once a smooth Helmholtz free energy is specified, organized to emphasize the role of the PDEL variational conjugacy.

Let a(ρ,θ,ξ) be the Helmholtz free energy per mass, where ρ is the mass density, θ the absolute temperature and ξ denotes additional internal variables. The corresponding free energy per reference volume is

(20) ϕ(ρ,θ,ξ)=ρa(ρ,θ,ξ).

In the PDEL action, the purely thermal storage at a material point enters through the combination

ϕ(ρ,θ,ξ)+θs.

As shown in Section 2, variations in the temperature at fixed (ρ,ξ) act only on this block, and the stationarity condition

(21) $$\delta_{\theta }\left[\phi (\rho ,\theta ,\xi )+\theta s\right]=0$$

yields, for arbitrary δθ,

(22) $$\left(\partial_{\theta }\phi \left(\rho ,\theta ,\xi \right)+s\right)\;\delta \theta =0\;⟹\;s\left(\rho ,\theta ,\xi \right)=-\partial_{\theta }\phi \left(\rho ,\theta ,\xi \right).$$

Dividing by ρ and using ϕ=ρa with ρ independent of θ at fixed (ρ,ξ) gives the mass-specific relation

(23) $$s\left(\rho ,\theta ,\xi \right)=-\partial_{\theta }a\left(\rho ,\theta ,\xi \right).$$

Thus the PDEL temperature variation reproduces the Helmholtz conjugacy $s=-\partial_{\theta }a$ familiar from classical continuum thermodynamics [6,12].

The pressure *p* follows from the standard Helmholtz identity. Matching the coefficients of dρ in the Gibbs relation yields the Helmholtz pressure identity [8]

(24) $$p\left(\rho ,\theta ,\xi \right)=\rho^{2}\;\partial_{\rho }a\left(\rho ,\theta ,\xi \right).$$

The heat capacities arise directly from the PDEL temperature form. From $s=-\phi_{\theta }$,

(25) $$\theta \;\dot{s}=\theta \;\partial_{\theta }s\;\dot{\theta }+\cdots =-\theta \;\partial_{\theta \theta }\phi \left(\theta ,\xi \right)\;\dot{\theta }+\cdots ,$$

so that the volumetric heat capacity is

(26) $$C\left(\theta ,\xi \right):=-\theta \;\partial_{\theta \theta }\phi \left(\theta ,\xi \right)\;(\mathrm{per}\;\mathrm{volume}).$$

Similarly, at the mass-specific level,

(27) $$c_{v}\left(\rho ,\theta ,\xi \right):=-\theta \;\partial_{\theta \theta }a\left(\rho ,\theta ,\xi \right)\;(\mathrm{per}\;\mathrm{mass}).$$

These expressions coincide with the standard continuum definitions once a smooth free energy is specified [6,12].

Further canonical identities (Gibbs free energy, response functions, and an ideal-gas example) are reported in Appendix A.

### 3.3. Heat Conduction: Fourier and Cattaneo–Vernotte

In the Fourier case the reference heat flux is taken as $q_{0}=-\;k\left(\theta \right)\;\nabla \theta$ with k(θ)≥0. Inserting this into Equation (11) and using $s=-\partial_{\theta }\phi \left(\theta ,Z\right)$ with $\partial_{\theta Z}\phi =0$ (separable thermal part) gives $\theta \;\dot{s}=C\left(\theta \right)\;\dot{\theta }$ and $\nabla \cdot q_{0}=-\;\nabla \cdot \left(k(\theta )\nabla \theta \right)$. Therefore,

(28) $$C\left(\theta \right)\;\dot{\theta }=\nabla \cdot \left(k(\theta )\;\nabla \theta \right)+\sum_{\alpha }\Phi_{\alpha }+r_{0}.$$

When no other channel is active ($\sum_{\alpha }\Phi_{\alpha }=0$), Equation (28) is precisely the Fourier heat equation in the reference frame [6]. In Clausius form Equation (11), the conductive production is $q_{0}\cdot \nabla \left(1/\theta \right)=k\left(\theta \right){\left|\nabla \theta \right|}^{2}/\theta^{2}\geq 0$, so that $\dot{s}=k\left(\theta \right){\left|\nabla \theta \right|}^{2}/\theta^{2}+\left(1/\theta \right)\sum_{\alpha }\Phi_{\alpha }+r_{0}/\theta \geq 0$.

To capture finite-speed heat signals, introduce a flux internal variable h(X,t) and identify the reference flux with it, $q_{0}=h$. Assign the channel co-force

(29) $$Y_{h}=\tau \;\dot{h}+h+K\;\nabla \theta ,\;\Phi_{h}=Y_{h}\cdot \dot{h}\geq 0,$$

where τ>0 is a relaxation time and *K* is positive definite. The weak balance then yields the constitutive relation

(30) $$\tau \;\dot{h}+h+K\;\nabla \theta =0,$$

which is the Cattaneo–Vernotte law [39,40,44]. With $\nabla \cdot q_{0}=\nabla \cdot h$ and Equation (11),

(31) $$C\left(\theta \right)\;\dot{\theta }=-\;\nabla \cdot h+\Phi_{h}+r_{0}.$$

Eliminating h gives a hyperbolic heat equation: differentiate Equation (31) in time and use $\dot{h}=-\left(h+K\nabla \theta \right)/\tau$ from Equation (30) to obtain

(32) $$\tau \;C\left(\theta \right)\;\ddot{\theta }+C\left(\theta \right)\;\dot{\theta }=\nabla \cdot \left(K\;\nabla \theta \right)+\tau \;{\dot{r}}_{0}+(\mathrm{terms}\;\mathrm{from}\;{\dot{\Phi }}_{h}),$$

which reduces to the classical telegraph-type form when C,K,τ are constant and sources vary slowly. In the Clausius balance the two contributions to entropy production are h·∇(1/θ) and $\Phi_{h}/\theta$, both nonnegative once Equation (30) is enforced.

### 3.4. Diffusion, Chemical Reactions and Electrochemistry

This section instantiates the general framework on diffusion, chemical reactions and electrochemical couplings, and then composes these channels with quasi-electrostatic closure. The aim is to show how each mechanism enters as a modular, nonnegative production consistent with classical nonequilibrium thermodynamics and gradient-flow structures [6,9,22,30,32].

For *N* species concentrations are denoted by $c_{i}\left(X,t\right)$ and the free energy density is $\phi (\theta ,\left\{c_{i}\right\})$. The chemical potentials are

(33) $$\mu_{i}\left(\theta ,\left\{c_{j}\right\}\right):=\frac{\partial \phi (\theta ,\left\{c_{j}\right\})}{\partial c_{i}},\;i=1,\ldots ,N,$$

and the mobility matrix $M\left(\theta ,\left\{c_{j}\right\}\right)=\left(M_{ij}\right)$ is assumed symmetric positive semidefinite. The chemical affinities are

(34) $$A_{r}\left(\theta ,\left\{c_{j}\right\}\right):=-\sum_{i=1}^{N}\nu_{ir}\;\mu_{i}\left(\theta ,\left\{c_{j}\right\}\right),$$

so that positive $A_{r}$ favors the forward reaction in the sense of De Groot–Mazur [9]. The total instantaneous production is decomposed as

(35) $$\Phi :=\Phi_{\mathrm{diff}}+\Phi_{\mathrm{chem}}+\Phi_{\mathrm{Joule}}+\cdots \geq 0,$$

and each contribution enters the entropy/heat balance as a positive source.

#### 3.4.1. Diffusion Channel

Species balance reads

(36) $$\dot{c}+\nabla \cdot j=r_{c},$$

and the constitutive closure is taken as a gradient-flow (Fick/Onsager) law

(37) j=−M(θ,c)∇μ,

with the corresponding diffusion production

(38) $$\Phi_{\mathrm{diff}}:=-\;j\cdot \nabla \mu =\nabla \mu^{⊤}M\left(\theta ,c\right)\;\nabla \mu \geq 0.$$

In the multispecies case,

(39) $$j_{i}=-\sum_{j=1}^{N}M_{ij}\left(\theta ,\left\{c\right\}\right)\;\nabla \mu_{j},\;i=1,\ldots ,N,$$

and

(40) $$\Phi_{\mathrm{diff}}=\sum_{i=1}^{N}j_{i}\cdot \nabla \mu_{i}=\nabla \mu^{⊤}M\;\nabla \mu \geq 0.$$

**Figure 1:**
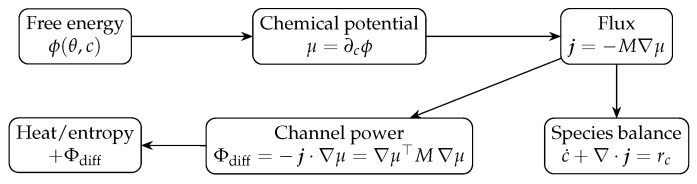
Diffusion channel (Fick/Onsager) and entropy production in PDEL. Free energy ϕ(θ,c) defines $\mu =\partial_{c}\phi$; with flux j=−M∇μ (M⪰0), the channel power $\Phi_{\mathrm{diff}}=-\;j\cdot \nabla \mu =M\;{\left|\nabla \mu \right|}^{2}\geq 0$ enters the heat/entropy balance as a positive source, and the species balance is $\dot{c}+{\mathrm{Div}}_{X}j=r_{c}$.

The process for the diffusion channel is depicted in Figure 1.

Substituting Equation (37) into Equation (36) gives species balance equation:

(41) $$\dot{c}-\nabla \cdot \left(M(\theta ,c)\;\nabla \mu \right)=r_{c}.$$

In the multispecies case,

(42) $${\dot{c}}_{i}-\nabla \cdot \left(\sum_{j=1}^{N}M_{ij}\;\nabla \mu_{j}\right)=r_{c_{i}},\;i=1,\ldots ,N.$$

From the entropy/heat balance supplied by the action,

(43) $$\theta \;\dot{s}+\nabla \cdot q_{0}-\Phi =r_{0},$$

With only diffusion active ($\Phi =\Phi_{\mathrm{diff}}$), the separable thermal part Equation (6) and Fourier’s law yield the temperature equation (see Appendix B.1).

(44) $$C\left(\theta \right)\;\dot{\theta }=\nabla \cdot \left(k(\theta )\;\nabla \theta \right)+\Phi_{\mathrm{diff}}+r_{0}=\nabla \cdot \left(k(\theta )\;\nabla \theta \right)+\nabla \mu^{⊤}\;M\left(\theta ,c\right)\;\nabla \mu +r_{0}.$$

#### 3.4.2. Chemical Reaction Channel

For reactions r=1,…,R with rates $R_{r}$ and stoichiometry $\nu_{ir}$, the species balances read

(45) $${\dot{c}}_{i}+\nabla \cdot j_{i}=\sum_{r=1}^{R}\nu_{ir}\;R_{r}+r_{c_{i}},\;i=1,\ldots ,N.$$

The affinity is defined by Equation (34), $A_{r}=-\sum_{i=1}^{N}\nu_{ir}\mu_{i}$ [9]. Near equilibrium one admits linear kinetics,

(46) $$R_{r}=\sum_{s=1}^{R}L_{rs}\left(\theta ,\left\{c_{j}\right\}\right)\;A_{s},\;L=\left(L_{rs}\right)\;\mathrm{symmetric},L⪰0,$$

which guarantees nonnegative reaction production [9,22]

(47) $$\Phi_{\mathrm{chem}}:=\sum_{r=1}^{R}A_{r}\;R_{r}=A^{⊤}L\;A\geq 0,\;A:={\left(A_{1},\ldots ,A_{R}\right)}^{⊤}.$$

With only reactions active, the entropy/heat balance Equation (43) gives

(48) $$C\left(\theta \right)\;\dot{\theta }=\nabla \cdot \left(k(\theta )\;\nabla \theta \right)+\Phi_{\mathrm{chem}}+r_{0}=\nabla \cdot \left(k(\theta )\;\nabla \theta \right)+\sum_{r=1}^{R}A_{r}\;R_{r}+r_{0}.$$

#### 3.4.3. Reaction–Diffusion–Heat (Modular Coupling)

Combining Equation (39) with (46), the total instantaneous production is,

(49) $$\Phi =\Phi_{\mathrm{diff}}+\Phi_{\mathrm{chem}}\geq 0,$$

and enters heat as +Φ, entropy production as Φ/θ. Equation (45) becomes

(50) $${\dot{c}}_{i}-\nabla \cdot \left(\sum_{j=1}^{N}M_{ij}\;\nabla \mu_{j}\right)=\sum_{r=1}^{R}\nu_{ir}\;R_{r}+r_{c_{i}},\;i=1,\ldots ,N.$$

With Equation (43), one obtains

(51) $$C\left(\theta \right)\dot{\theta }=\nabla \cdot \left(k(\theta )\nabla \theta \right)+\sum_{i=1}^{N}j_{i}\cdot \nabla \mu_{i}+\sum_{r=1}^{R}A_{r}R_{r}+r_{0}.$$

Equations (50) and (51) provide a compact reaction–diffusion–heat system consistent with classical nonequilibrium thermodynamics and metric gradient-flow structures.

A coupled electrochemistry–diffusion–reaction–heat example (battery half-cell), including Joule heating and optional electrostatics closure, is given in Appendix B.2.

The above channels illustrate the modular structure: productions add as $\Phi =\Phi_{\mathrm{diff}}+\Phi_{\mathrm{chem}}+\cdots \geq 0$, enter the entropy/heat balance as a positive source, and appear with the opposite sign in the corresponding channel balances.

### 3.5. Physical Interpretation

Although the oscillator and transport examples are presented in an abstract channel form, the physical content is simple: each dissipative mechanism is treated as a separate power channel whose instantaneous dissipation $\Phi_{\alpha }\geq 0$ is deposited once into the entropy/heat balance and appears with the opposite sign in the corresponding channel equation.

For the 1-DOF oscillator, $\Phi =c\;{\dot{u}}^{2}$ is the mechanical power irreversibly converted into heat, so the bookkeeping immediately fixes the sign and magnitude of the thermal source term and prevents counting the same loss both as a damping force and as an additional heat source. For transport, the same rule assigns $\Phi_{\mathrm{diff}}=\nabla \mu^{⊤}M\nabla \mu$ (and, if present, $\Phi_{\mathrm{chem}}=A^{⊤}LA$, $\Phi_{\mathrm{Joule}}=\sigma_{\mathrm{el}}{\left|E\right|}^{2}$, etc.) as additive, nonnegative heat sources, providing a clear checklist when multiple mechanisms are coupled. This single-entry audit is equally useful in numerics: discrete updates can be designed so that each channel contributes one nonnegative increment to the discrete entropy production and one matching decrement in its own balance, yielding an algorithmic energy/entropy audit that mirrors the continuum identities.

## 4. Relation and Mapping to Onsager, EDP, and GENERIC

Many of these target frameworks can, in principle, represent the same evolution equations once suitable mobilities, dissipation potentials, or brackets are prescribed. In this sense, the present PDEL is not meant as “another gradient-flow representation” of a given PDE. Its distinctive element is action-level: the entropy/heat audit is embedded through the history integral and the associated upper-limit (time-relabeling) variation, and the weak channel power balance enforces a single-entry placement of each production across the entropy/heat balance and the corresponding channel equation. The mappings below therefore highlight equivalences at the level of closures, while emphasizing that PDEL fixes the accounting and sign conventions by construction.

This section makes explicit how the present variational construction (i) reproduces linear nonequilibrium closures in the sense of Onsager, (ii) specializes to the energy dissipation principle (EDP) and to metric gradient flows when the dissipation potential is convex, and (iii) corresponds to the dissipative sector of GENERIC/metriplectic formulations, while separating reversible and irreversible parts. The key hinge is the sign placement supplied by the action: every channel with rate/force pair $\left({\dot{Z}}_{\alpha },Y_{\alpha }\right)$ contributes

$$\Phi_{\alpha }\;=Y_{\alpha }\;{\dot{Z}}_{\alpha }\geq 0$$

positively to heat/entropy (via the upper-limit variation in the history term) and negatively in the tested channel equation (via weak power balance). This consistent energy accounting pins the sign structure without multipliers or Rayleigh potentials and prevents counting the same dissipation twice [6,9]. A mapping from PDEL to Onsager, EDP, and GENERIC is summarized in Table 1.

### 4.1. Onsager (Near Equilibrium): Linear Closures and Reciprocity

In linear nonequilibrium thermodynamics one identifies sets of forces *F* and fluxes (rates) *J* related by

(52) $$J=L\;F,\;L=L^{⊤}⪰0,$$

with entropy production density σ=F·J≥0 and the *reciprocity* $L=L^{⊤}$ [9,22,23,24]. Cross-effects appear as off-diagonal entries of *L*.

Choose for a given channel the identifications

(53) $$J=\dot{Z},\;F=Y,\;\sigma =\Phi =Y:\dot{Z}.$$

If the constitutive closure is linear near equilibrium, take *L* in Equation (52) so that $\dot{Z}=LY$ (or equivalently $Y=L^{-1}\dot{Z}$ when *L* is invertible). Then the production is

(54) $$\Phi =Y:\dot{Z}=Y:\left(L\;Y\right)=Y^{⊤}L\;Y\geq 0,$$

and cross-effects are encoded in *L*. Substituting Equation (54) into Equation (10) deposits Φ/θ into entropy production. The tested channel equation E+Y=0 ensures the same Φ appears with sign −Φ in the channel, agreeing with the single-entry structure.

Let $\mu_{i}=\partial_{c_{i}}\phi$ be chemical potentials. With the Onsager closure $j_{i}=-\sum_{j}M_{ij}\nabla \mu_{j}$ (here J↔j and F↔∇μ), the production of multicomponent diffusion

(55) $$\Phi_{\mathrm{diff}}=\sum_{i}j_{i}\cdot \nabla \mu_{i}={\left(\nabla \mu \right)}^{⊤}M\left(\nabla \mu \right)\geq 0$$

matches the Onsager form with L=M [6,9]. Equation (10) puts $+\Phi_{\mathrm{diff}}/\theta$ in entropy and Equation (15) puts $-\Phi_{\mathrm{diff}}$ in the tested diffusion channel.

**Comment on reciprocity.** Symmetry of *L* can be imposed at the closure level or derived from microreversibility assumptions close to equilibrium [22,23,24]. The present mapping requires only L⪰0 to guarantee Φ≥0 and does not invoke multipliers in the action.

### 4.2. EDP/Metric Gradient Flows: Convex Potentials and Fenchel–Young Identity

In the energy dissipation principle one prescribes an energy E(Z) and a convex, proper, lower semicontinuous dissipation potential $R(\dot{Z})$, with convex dual $R^{*}\left(Y\right):=\sup_{V}\left\{\;Y:V-R\left(V\right)\;\right\}$ [30,32,33]. The EDP balance relies on the Fenchel–Young identity

(56) $$R\left(\dot{Z}\right)+R^{*}\left(Y\right)\geq Y:\dot{Z},\;\mathrm{with}\;\mathrm{equality}\;\mathrm{iff}\;Y\in \partial_{\dot{Z}}R\left(\dot{Z}\right)⟺\dot{Z}\in \partial_{Y}R^{*}\left(Y\right).$$

Select a purely dissipative setting (no inertia) so that the reversible residual is $E=\partial_{Z}\phi$ with ϕ the stored/free energy. Choose the channel co-force

(57) $$Y\in \partial_{\dot{Z}}R\left(\dot{Z}\right),$$

that is, the dissipative force is the subgradient of R. The weak balance E+Y=0 then reads

(58) $$0\in \partial_{\dot{Z}}R\left(\dot{Z}\right)+\partial_{Z}\phi \left(Z\right),$$

which is the standard EDP stationarity (a differential inclusion in general). Testing Equation (58) by $\dot{Z}$ and invoking Equation (56) gives

(59) $$E:\dot{Z}+R\left(\dot{Z}\right)+R^{*}\left(Y\right)=0,$$

because $Y:\dot{Z}=R\left(\dot{Z}\right)+R^{*}\left(Y\right)$ at equality. Using the PDEL power identity $E:\dot{Z}+\Phi =0$ from Equation (15), we identify

(60) $$\Phi =R\left(\dot{Z}\right)+R^{*}\left(Y\right)\geq 0.$$

Substituting Equation (60) into the entropy balance Equation (10) yields the EDP energy–entropy structure within the same action, with no separate Clausius–Duhem inequality required [32,33].

If $R\left(\dot{Z}\right)=\frac{1}{2}\;{\dot{Z}}^{⊤}M\;\dot{Z}$ with M(Z) symmetric positive definite, then $Y=\partial_{\dot{Z}}R=M\;\dot{Z}$ and Equation (58) reduces to

(61) $$M\;\dot{Z}+\partial_{Z}\phi =0\;⟺\;\dot{Z}=-\;M^{-1}\partial_{Z}\phi ,$$

the metric gradient flow of ϕ in the metric induced by $M^{-1}$ [30,32]. The production becomes

(62) $$\Phi =Y:\dot{Z}={\dot{Z}}^{⊤}M\;\dot{Z}={\left(\partial_{Z}\phi \right)}^{⊤}M^{-1}\left(\partial_{Z}\phi \right)\geq 0,$$

and Equation (10) deposits Φ/θ into entropy. Thus, EDP and metric gradient flows are embedded in PDEL by the single choice Equation (57).

**Remark on nonquadratic and rate-independent cases.** When R is nonquadratic (e.g., power law, Bingham, or rate-independent dissipation), the inclusion Equation (58) still holds with $Y\in \partial R(\dot{Z})$ and $\Phi =R+R^{*}$ remains nonnegative by convex duality [30,32].

### 4.3. GENERIC/Metriplectic: Dissipative Sector and Compatibility

In GENERIC one combines a Poisson (Hamiltonian) bracket with a symmetric dissipative bracket acting on functionals of the state Z to evolve energy H(Z) and entropy S(Z) under the compatibility (degeneracy) conditions [25,27,28]:

(63) $$\dot{Z}=L\left(Z\right)\;\nabla H\left(Z\right)+M\left(Z\right)\;\nabla S\left(Z\right),$$

where L(Z) is antisymmetric and M(Z) symmetric positive semidefinite, subject to

(64) L(Z)∇S(Z)=0,M(Z)∇H(Z)=0.

Then $\dot{H}=\nabla H:M\;\nabla S=0$ and $\dot{S}=\nabla S:M\;\nabla S\geq 0$.

Consider the PDEL channel equation E+Y=0 with the quadratic metric choice $Y=M\left(Z\right)\;\dot{Z}$. If inertia is neglected and $E=\partial_{Z}\phi$ with ϕ the stored energy, the channel equation gives $M\left(Z\right)\;\dot{Z}+\partial_{Z}\phi =0$, i.e., the gradient flow Equation (61). Identifying ϕ=H and viewing *S* as the entropy controlled by Equation (10), the dissipation power is $\Phi ={\dot{Z}}^{⊤}M\;\dot{Z}\geq 0$ as in Equation (62). Since the production enters only through $Y:\dot{Z}$, reversible flows leave Equation (10) unaffected.

If a Poisson contribution is present in the reversible residual *E* (for instance, through a Hamiltonian term in T−ϕ that generates L(Z)∇H in the Euler-Lagrange part), the dissipative part $Y=M\;\dot{Z}$ still yields $\Phi ={\dot{Z}}^{⊤}M\;\dot{Z}\geq 0$, and the entropy balance Equation (10) remains valid because the history integrand collects only dissipative productions and heat divergence. This is consistent with the GENERIC degeneracy M∇H=0 in the sense that the dissipative channel does not inject or remove Hamiltonian energy [25,27,28].

Thus, PDEL recovers the metric (symmetric) sector of GENERIC and allows the reversible (Poisson) sector to be superposed without altering the entropy production. Recent geometric formulations (e.g., contact structures for nonequilibrium systems) are compatible with this split as well [35].

Testing $E+M\dot{Z}=0$ by $\dot{Z}$ yields $E:\dot{Z}+{\dot{Z}}^{⊤}M\dot{Z}=0$, that is,

(65) $$E:\dot{Z}+\Phi =0,\;\Phi ={\dot{Z}}^{⊤}M\dot{Z}\geq 0,$$

which is exactly the power identity Equation (15). Substituting Φ into Equation (10) gives $\theta \dot{s}+\nabla \cdot q_{0}-{\dot{Z}}^{⊤}M\dot{Z}=r_{0}$, or upon division by θ the nonnegative entropy production ${\dot{s}}_{\mathrm{prod}}={\dot{Z}}^{⊤}M\dot{Z}/\theta$.

## 5. Conclusions

A single path-dependent action is introduced whose stationarity yields both the entropy/heat balance and a channel power balance identity, thereby avoiding Lagrange multipliers and Rayleigh potentials. The modular “channel” architecture unifies Fourier and non-Fourier heat conduction, diffusion, and viscous mechanics, and extends to coupled thermo-mechanical and transport processes. The calculus ensures consistent energy accounting of dissipation and provides a concise route to the entropy/heat balance. It also offers a clean spatial reference mapping for continuum fields. Together, these features simplify model assembly and make entropy accounting clearer in practice.

The analysis assumes local balances and sufficient regularity of paths to justify tangential variations and integrations by parts. Nonlocal kernels, measure-valued solutions, and full well-posedness theory are not addressed. Rate-independent evolutions with jumps require a careful measure-theoretic extension, and stochastic forcing and constraints are likewise outside the present scope.

The PDEL structure provides a compact mapping to standard nonequilibrium formalisms. With rates identified as fluxes and co-forces as thermodynamic forces, it recovers Onsager-type relations and nonnegative production. With convex dissipation potentials, it reproduces the energy dissipation principle and metric gradient flows, while symmetric mobility operators correspond to the dissipative sector of GENERIC/metriplectic dynamics and its reversible–dissipative split.

Beyond Fourier/Fick closures, relaxation-type channels (Cattaneo, Guyer–Krumhansl) and viscoelastic/viscoplastic responses fit by declaring their rate-force pairs and depositing their non-negative production into the entropy audit. Structure-preserving time discretizations (e.g., minimizing-movement schemes) that inherit the one-way energy deposition are a potential direction for numerical analysis, alongside extensions to nonlocal and stochastic PDEL formulations.

## Figures and Tables

**Table 1:** Relation of the PDEL channel structure to Onsager, EDP/gradient flows, and GENERIC/metriplectic formalisms.

Framework	Identification/Mapping	Representative Expression
PDEL channel	$\left({\dot{Z}}_{\alpha },Y_{\alpha }\right)$, $\Phi_{\alpha }\;\geq \;0$	$\Phi_{\alpha }\;=\;Y_{\alpha }{\dot{Z}}_{\alpha }\geq 0,\theta \dot{s}\;+\;\nabla \;\;\cdot \;q_{0}-\sum_{\alpha }\;\Phi_{\alpha }\;=\;r_{0}$
Onsager	$\dot{Z}=J$ (flux), Y=F	$J=LF,\;L=L^{⊤}⪰0,\;\Phi =F\;\cdot \;J\geq 0$
EDP/gradient flows	$R(\dot{Z})$, $Y\in \partial_{\dot{Z}}R$	$0\;\in \;\partial_{\dot{Z}}R\left(\dot{Z}\right)\;+\;\partial_{Z}\phi \left(Z\right),\dot{Z}\;=\;-M\partial_{Z}\phi$
EDP (energy dissipation)	$R^{*}\left(Y\right)$, with $Y\;=\;-\partial_{Z}\phi$	$\frac{d}{dt}\phi \left(Z\right)+R\left(\dot{Z}\right)+R^{*}\left(-\partial_{Z}\phi \right)\;=\;0$
GENERIC/metriplectic	$M\;=\;M^{⊤}⪰0$, Y=δS/δZ	$\dot{Z}=M\;\frac{\delta S}{\delta Z},\;\dot{S}=\langle \frac{\delta S}{\delta Z},M\frac{\delta S}{\delta Z}\rangle \;\geq \;0$
GENERIC(full split)	$L\;=\;-L^{⊤}$ (Poisson), $M\;=\;M^{⊤}$	$\dot{Z}=L\frac{\delta E}{\delta Z}+M\;\frac{\delta S}{\delta Z}$

## Data Availability

The data presented in this study are available on request from the corresponding author.

## Abbreviations

Summary of notation.

**Symbol**	**Meaning**	**Notes**
*t*	time	$t\in [t_{0},T]$
$t_{0},\;T$	initial/final time	in the action
X	reference coordinate	X∈Ω
Ω	reference domain	
∇(·), ∇·(·)	gradient/divergence	w.r.t. X
$\dot{\left(\cdot \right)}$	material time derivative	
δ(·)	virtual variation	
δt	tangential time variation	time relabeling/upper-limit rule
Z	generalized state variables	components $Z_{\alpha }$
$Z_{\alpha }$	channel variable	indexed by α
${\dot{Z}}_{\alpha }$	channel rate	
$Y_{\alpha }$	channel co-force	paired with ${\dot{Z}}_{\alpha }$
$\Phi_{\alpha }$	channel production	$\Phi_{\alpha }=Y_{\alpha }{\dot{Z}}_{\alpha }\geq 0$
Φ	total production	$\Phi =\sum_{\alpha }\Phi_{\alpha }$
*K*	kinetic energy density	e.g., $K=\frac{1}{2}{\dot{Z}}^{⊤}M\dot{Z}$
*M*	mass matrix/operator	symmetric positive definite
ϕ(θ,Z)	Helmholtz free-energy density	per reference volume
θ	absolute temperature	θ>0
*s*	entropy density	per reference volume
*e*	internal energy density	e=ϕ+θs
C(θ,Z)	volumetric heat capacity	$C=-\theta \;\partial_{\theta \theta }\phi \geq 0$
$q_{0}$	reference heat flux	Fourier: $q_{0}=-k\nabla \theta$
k(θ)	thermal conductivity	k≥0
$r_{0}$	external heating	per reference volume
*D*	history integrand	$D=\nabla \;\cdot q_{0}-r_{0}-\Phi$
S[·]	PDEL action functional	see Equation (1)
$c_{i}$	concentration of species *i*	i=1,…,N
$j_{i}$	species flux	
$\mu_{i}$	chemical potential	$\mu_{i}=\partial \phi /\partial c_{i}$
$M_{ij}$	mobility matrix (diffusion)	symmetric, positive semidefinite
$\nu_{ir}$	stoichiometric coefficient	reaction *r*
$R_{r}$	reaction rate	
$A_{r}$	affinity	$A_{r}=-\sum_{i}\nu_{ir}\mu_{i}$
$L_{rs}$	Onsager matrix (kinetics)	symmetric, positive semidefinite

## Appendix A. Additional Canonical Thermodynamic Identities

It is often convenient to work with the Gibbs free energy per mass,

(A1) $$g\left(p,\theta ,\xi \right):=a\left(\rho ,\theta ,\xi \right)+\frac{p}{\rho },\;v:=\rho^{-1},$$

that is, g=a+pv. This is the Legendre transform of *a* with respect to (v,θ,ξ) and treating *g* as a function of (p,θ,ξ),

(A2) dg=da+vdp+pdv.

Using e/ρ=a+θs and the Gibbs relation again, one finds

da=−sdθ−pdv+(workofξ),

so that

(A3) dg=−sdθ+vdp+⋯.

Reading off the coefficients at fixed ξ gives the classical conjugacies

(A4) $$s\left(p,\theta ,\xi \right)=-\partial_{\theta }g\left(p,\theta ,\xi \right),\;v\left(p,\theta ,\xi \right)=\partial_{p}g\left(p,\theta ,\xi \right).$$

The thermal expansion coefficient α and isothermal compressibility $\kappa_{T}$ can now be expressed in terms of derivatives of *g*. By definition [8,52],

(A5) $$\alpha :=\frac{1}{v}{\left(\frac{\partial v}{\partial \theta }\right)}_{p},\;\kappa_{T}:=-\frac{1}{v}{\left(\frac{\partial v}{\partial p}\right)}_{\theta }.$$

Using $v=g_{p}$, one obtains

(A6) $$\alpha =\frac{1}{v}\;\partial_{\theta }v{|}_{p}=\frac{1}{v}\;\partial_{\theta p}g=-\frac{1}{v}\;\partial_{\theta p}g,$$

where the last equality incorporates the sign convention in *g*. Likewise,

(A7) $$\kappa_{T}=-\frac{1}{v}\;\partial_{p}v{|}_{\theta }=-\frac{1}{v}\;\partial_{pp}g=\frac{1}{v}\;\partial_{pp}g,$$

again up to sign conventions consistent with standard texts [8,52].

The specific heat at constant pressure is

(A8) $$c_{p}:=\theta {\left(\frac{\partial s}{\partial \theta }\right)}_{p,\xi }=-\theta \;\partial_{\theta \theta }g\left(p,\theta ,\xi \right),$$

while $c_{v}$ is already given by $c_{v}=-\theta a_{\theta \theta }$. Using the Maxwell relation

$${\left(\frac{\partial s}{\partial p}\right)}_{\theta }=-{\left(\frac{\partial v}{\partial \theta }\right)}_{p}=-\alpha v,$$

and changing variables between (ρ,θ) and (p,θ), one recovers the standard identity [8,52]

(A9) $$c_{p}-c_{v}=\frac{\theta \;\alpha^{2}}{\rho \;\kappa_{T}},$$

which shows how thermoelastic coupling, encoded in α and $\kappa_{T}$, controls the gap between $c_{p}$ and $c_{v}$. All of these response functions are generated within the PDEL framework once a smooth *a* or *g* is prescribed.

As a concrete example, consider an ideal gas with Helmholtz free energy per mass [8,52]

(A10) $$a\left(\rho ,\theta \right)=c_{v}\;\theta \left[1-\ln\left(\frac{\theta }{\theta_{*}}\right)\right]+R_{s}\;\theta \;\ln\left(\frac{\rho }{\rho_{*}}\right),$$

where $\theta_{*}$ and $\rho_{*}$ are reference scales, $c_{v}>0$ is the constant specific heat at volume, and $R_{s}$ is the specific gas constant.

From $s=-a_{\theta }$ one obtains

(A11) $$s\left(\rho ,\theta \right)=c_{v}\ln\left(\frac{\theta }{\theta_{*}}\right)-R_{s}\ln\left(\frac{\rho }{\rho_{*}}\right),$$

and hence

$$\frac{e}{\rho }=a+\theta s=c_{v}\;\theta ,$$

so that $e=\rho c_{v}\theta$ as expected. The pressure follows from the Helmholtz identity:

(A12) $$p\left(\rho ,\theta \right)=\rho^{2}\;\partial_{\rho }a\left(\rho ,\theta \right)=\rho^{2}\left(R_{s}\;\theta \;\frac{1}{\rho }\right)=R_{s}\;\rho \;\theta ,$$

which is the ideal-gas law. The specific volume is $v=1/\rho =R_{s}\theta /p$, and direct differentiation at fixed *p* and fixed θ gives

(A13) $$\alpha =\frac{1}{v}{\left(\frac{\partial v}{\partial \theta }\right)}_{p}=\frac{1}{\theta },\;\kappa_{T}=-\frac{1}{v}{\left(\frac{\partial v}{\partial p}\right)}_{\theta }=\frac{1}{p}.$$

Moreover, $a_{\theta \theta }=-c_{v}/\theta$ leads to $c_{v}=-\theta a_{\theta \theta }=c_{v}$ (constant) and $c_{p}=c_{v}+R_{s}$ as usual [8,52].

In the PDEL temperature balance (per current volume),

(A14) $$\rho c_{v}\;\dot{\theta }=\nabla \cdot \left(k\nabla \theta \right)-p\;\nabla \cdot u+\sum_{\alpha }\Phi_{\alpha }+r,$$

the reversible term −p∇·u arises from the mixed derivative of the free energy with respect to (ρ,θ) (equivalently, from the pv part of *g*) and represents compressive heating/cooling. In the Clausius form of the PDEL entropy balance, this term belongs to the reversible part of $\theta \dot{s}$ and does not contribute to the entropy production; only the conduction and channel productions $\Phi_{\alpha }$ appear on the production side [6,12]. This example illustrates how PDEL reproduces the standard thermodynamics of an ideal gas, including compressive heating, within the same variational structure that also governs irreversible processes.

## Appendix B. Auxiliary Derivations for Section 3.4

### Appendix B.1. From the Entropy/Heat Balance to the Temperature Equation

Using the separable thermal contribution $\phi_{\mathrm{th}}\left(\theta \right)$ in Equation (6) and Fourier’s law $q_{0}=-k\left(\theta \right)\nabla \theta$, one has

(A15) $$\begin{array}{cc} \theta \;\dot{s} & =\theta \frac{d}{dt}\left(-\;a^{\prime }\left(\theta \right)\right)=-\theta \;a^{\prime \prime }\left(\theta \right)\;\dot{\theta }=C\left(\theta \right)\;\dot{\theta }, \end{array}$$

(A16) $$\begin{array}{cc} \nabla \cdot q_{0} & =\nabla \cdot \left(-k(\theta )\;\nabla \theta \right)=-\nabla \cdot \left(k(\theta )\;\nabla \theta \right). \end{array}$$

Substituting this identity into Equation (43) yields Equation (44).

### Appendix B.2. Electrochemistry–Diffusion–Reaction–Heat Extension

Let φ(X,t) denote the electric potential, E=−∇φ the electric field, and i=σ(X,θ)E the conduction current with conductivity σ≥0. In a quasi-electrostatic regime,

(A17) ∇·i=0,

and, if desired, Poisson’s equation closes the potential,

(A18) $$-\;\nabla \cdot \left(\varepsilon \;\nabla \varphi \right)=F\sum_{i=1}^{N}z_{i}\;c_{i},$$

with permittivity ε, Faraday constant *F*, and valences $z_{i}$ [6]. The instantaneous Joule power is

(A19) $$\Phi_{\mathrm{Joule}}:=i\cdot E=\sigma \left(X,\theta \right)\;{\left|\nabla \varphi \right|}^{2}\geq 0.$$

Species transport and interfacial reactions are obtained as follows. Species $c_{i}$ move with fluxes

(A20) $$j_{i}=-\sum_{j=1}^{N}M_{ij}\left(\theta ,\left\{c\right\}\right)\;\nabla \mu_{j},$$

and undergo interfacial reactions indexed by *r* (e.g., Butler-Volmer-type kinetics), with affinities $A_{r}$ as in Equation (34) and rates $R_{r}$. The total production is

(A21) $$\Phi =\underset{\Phi_{\mathrm{Joule}}}{\underset{︸}{{\sigma \;|\nabla \varphi |}^{2}}}+\underset{\Phi_{\mathrm{diff}}}{\underset{︸}{\sum_{i=1}^{N}j_{i}\cdot \nabla \mu_{i}}}+\underset{\Phi_{\mathrm{chem}}}{\underset{︸}{\sum_{r=1}^{R}A_{r}R_{r}}}\geq 0.$$

Species balances read

(A22) $${\dot{c}}_{i}-\nabla \cdot \left(\sum_{j=1}^{N}M_{ij}\;\nabla \mu_{j}\right)=\sum_{r=1}^{R}\nu_{ir}\;R_{r}+r_{c_{i}},\;i=1,\ldots ,N.$$

Using Equation (43) with Φ from Equation (A21) and the separable thermal part Equation (6) gives the temperature equation:

(A23) $$C\left(\theta \right)\;\dot{\theta }=\nabla \cdot \left(k(\theta )\;\nabla \theta \right)+\sigma \;{\left|\nabla \varphi \right|}^{2}+\sum_{i=1}^{N}j_{i}\cdot \nabla \mu_{i}+\sum_{r=1}^{R}A_{r}R_{r}+r_{0}.$$

Dividing Equation (43) by θ shows that the local entropy production density is

(A24) $${\dot{s}}_{\mathrm{prod}}=\frac{1}{\theta }\;\left({\sigma \;|\nabla \varphi |}^{2}+\sum_{i=1}^{N}j_{i}\cdot \nabla \mu_{i}+\sum_{r=1}^{R}A_{r}R_{r}\right)\geq 0,$$

which is consistent with the Clausius-Duhem inequality in continuum thermodynamics [6,9]. If electrostatics is included variationally, one adds the reversible electrostatic energy $\frac{1}{2}\;\varepsilon \;{\left|\nabla \varphi \right|}^{2}$ to the free-energy part and varies φ; otherwise φ is prescribed by Equations (A17) and (A18). In both cases the same single-source heat/entropy audit follows from the action with a unique Φ.

The three channels — diffusion, chemical reaction, and Joule conduction—provide a modular collection of nonnegative productions. The species balances Equations (42), (45), (50) and (A22) follow standard conservation laws, whereas the constitutive closures Equations (39) and (46) ensure Φ≥0 in accordance with Onsager reciprocity and with the metric structure of gradient flows [9,22,30,32]. The heat equation Equations (44), (48), (51) and (A23) emerges from a single entropy/heat balance, placing all instantaneous productions on the right-hand side (positive in heat). The same productions appear with a negative sign in the tested channel equations, implementing single-entry power accounting. Limitations of the present demonstration include the separability assumption Equation (6) and the quasi-electrostatic setting; both can be relaxed with additional structure [6].
